# Real-Time Continuous Glucose Monitoring Shows High Accuracy within 6 Hours after Sensor Calibration: A Prospective Study

**DOI:** 10.1371/journal.pone.0060070

**Published:** 2013-03-28

**Authors:** Xiao-Yan Yue, Yi Zheng, Ye-Hua Cai, Ning-Ning Yin, Jian-Xin Zhou

**Affiliations:** Department of Critical Care Medicine, Beijing Tiantan Hospital, Capital Medical University, Beijing, China; University of Houston, United States of America

## Abstract

Accurate and timely glucose monitoring is essential in intensive care units. Real-time continuous glucose monitoring system (CGMS) has been advocated for many years to improve glycemic management in critically ill patients. In order to determine the effect of calibration time on the accuracy of CGMS, real-time subcutaneous CGMS was used in 18 critically ill patients. CGMS sensor was calibrated with blood glucose measurements by blood gas/glucose analyzer every 12 hours. Venous blood was sampled every 2 to 4 hours, and glucose concentration was measured by standard central laboratory device (CLD) and by blood gas/glucose analyzer. With CLD measurement as reference, relative absolute difference (mean±SD) in CGMS and blood gas/glucose analyzer were 14.4%±12.2% and 6.5%±6.2%, respectively. The percentage of matched points in Clarke error grid zone A was 74.8% in CGMS, and 98.4% in blood gas/glucose analyzer. The relative absolute difference of CGMS obtained within 6 hours after sensor calibration (8.8%±7.2%) was significantly less than that between 6 to 12 hours after calibration (20.1%±13.5%, p<0.0001). The percentage of matched points in Clarke error grid zone A was also significantly higher in data sets within 6 hours after calibration (92.4% versus 57.1%, p<0.0001). In conclusion, real-time subcutaneous CGMS is accurate in glucose monitoring in critically ill patients. CGMS sensor should be calibrated less than 6 hours, no matter what time interval recommended by manufacturer.

## Introduction

Epidemiologic data have shown that there is a high incidence of hyperglycemia in critically ill patients, and its occurrence is associated with adverse clinical outcome [1], [2]. On the other hand, intensive glucose control may carry the risk of inducing hypoglycemia [3], [4]. Recent study by The NICE-SUGAR Study Investigators suggested that both of moderate and severe hypoglycemia induced by intensive glucose control were associated with an increased risk of death in critically ill patients [5]. Although the appropriate target range of blood glucose in critically ill patients is inconclusive at present time, it has been widely accepted that accurate and timely measurement of blood glucose is essential in intensive care unit (ICU) setting, even in patients receiving a conventional glucose control protocol [6], [7].

There are currently two options for clinical glucose measurement in hospitalized patients: central laboratory devices (CLD) and point-of-care (POC) devices. Although CLD provides the most accurate results [8], it is not suitable for bedside glucose monitoring in ICU because of its slow turn-around time. POC devices are more commonly used for glucose monitoring in ICU patients [3]. POC handheld glucose analyzer with capillary blood sampling is originally designed for patient's home use as self-monitoring of blood glucose. Although handheld glucose analyzer can provide a fast bedside result, its accuracy in critically ill patients has been questioned [9], [10]. Another type of POC instrument commonly used in glucose monitoring in ICU is blood gas analyzer with function of glucose measurement [3]. It has been found that glucose measurements by blood gas/glucose analyzers located in ICU are more accurate than those by handheld glucose analyzers [11], [12]. The main disadvantage of glucose measurement by a blood gas/glucose analyzer is its intermittent and invasive nature. The limitation of glucose monitoring method in critical care settings may contribute to the discrepancy of results in glucose control studies, which may hamper the further investigation [13]. These issues have urged the need for real-time continuous glucose monitoring system (CGMS) devices [6].

For the past few years, several CGMS devices have been applied in critically ill patients [11], [14]–[26]. However, preliminary results of CGMS accuracy in ICU patients have been mixed, and seldom studies employed CLD serum glucose measurement as reference in accuracy investigation. Most CGMS devices measure subcutaneous interstitial glucose concentration by enzymatic glucose oxidase electrode, thus timed calibration of CGMS sensor by blood glucose measurement is required [27]. Up to now, there has been no study carried out to evaluate the influence of calibration method on CGMS accuracy. In present study, a real-time subcutaneous CGMS was used in adult critically ill patients to evaluate CGMS accuracy with standard CLD serum glucose measurement as reference. The purpose of this study was to determine the accuracy of CGMS, especially for the influence of calibration time on accuracy.

## Materials and Methods

### Ethics Statement

The study was performed in accordance with the Declaration of Helsinki, and study protocol was reviewed and approved by Research Ethic Committee in Beijing Tiantan Hospital, Capital Medical University (Beijing, China). Written informed consent was obtained from patients or their relatives.

### Study population and routine practice for glucose control

We carried out this prospective study in a 12-bed neurosurgical ICU in a 1000-bed university hospital, from January to April in 2012. Consecutive patients were screened and enrolled if they had hyperglycemia (blood glucose concentration greater than 10.0 mmol/L measured by ICU-based blood gas/glucose analyzer) at admission, and their expected lengths of stay in ICU were more than 48 hours. Exclusion criteria were patients younger than 18 years old, patients with hemoglobin concentration less than 100 g/L, patients admitted only for overnight postoperative monitoring, or patients in moribund and not likely to survive more than 24 hours. The demographic characteristics of enrolled patients were collected prospectively, including reasons for ICU admission, age, sex, history of diabetes mellitus, blood glucose concentration at admission, Acute Physiology And Chronic Health Evaluation (APACHE) II score on ICU admission, and use of insulin infusion during study period.

We employed a conventional glucose control protocol in our ICU [28], [29]. Continuous insulin infusion was initiated if the blood glucose concentration measured by ICU-based blood gas/glucose analyzer exceeded 11.1 mmol/L. The blood glucose target was set between 7.8 and 11.1 mmol/L. When the blood glucose concentration fell below 10.0 mmol/L, the insulin infusion was decreased. When the blood glucose fell below 7.8 mmol/L, the insulin infusion was stopped.

### Real-time CGMS

Glucose levels of enrolled patients were monitored by a real-time subcutaneous CGMS, the San MediTech's Dynamic Glucose Monitoring System (DGMS®, San Meditech Medical Technology Co., Ltd, Huzhou, Zhejiang, China). This system is composed of three parts: a disposable subcutaneous glucose sensor, a pager-sized monitor, and dynamic glucose analysis software for downloading stored data to a computer. The subcutaneous sensor contains an enzymatic glucose oxidase electrode connecting to the monitor by a cable. The values are displayed on the monitor as means of 16 glucose measurements over the last 3 minutes, allowing real-time continuous glucose monitoring. The range of glucose measurement by this CGMS is 1.7 to 25.0 mmol/L.

After patient recruitment, CGMS sensor was placed in the subcutaneous tissue on the left or right upper arm, and transparent tape was used to secure the sensor to the skin. After 3 hours warm-up period for CGMS, whole blood was sampled from a deep venous catheter, and blood glucose concentration was measured by a GEM Premier 3000 blood gas/glucose analyzer (Instrumentation Laboratory, Lexington, MA, USA) with the glucose-oxidase methods. This blood glucose value was used as the initial CGMS calibration. Subsequent calibrations were performed every 12 hours by using the same method. The GEM Premier 3000 blood gas/glucose analyzer was located in ICU. Sensor site was inspected by one of the investigators at least twice daily for signs of local irritation, infection, or bleeding. According to manufacturer's instruction manual, a CGMS sensor can be used up to 72 hours. Sensors were removed if the patient's glucose concentration had been stayed within target range for 24 hours, or in other cases the patient was transferred to another unit or died. The monitor can automatically detect sensor malfunction, which occurs because of low sensor current. If a sensor failed longer than 1 hour, the sensor was removed and a new sensor was inserted.

### Study protocol

Blood samples were obtained every 2 to 4 hours during study period. Approximately 2 ml of blood was withdrawn in a heparinized syringe (BD Preset™, LOT: 1263532, Becton Dickinson and Company, Plymouth, UK) from a deep venous catheter after 3 ml of blood was discarded. Additional samples were also collected at ICU physicians' own discretion for clinical need by using the same method. All blood samples were collected by one of the investigators.

The blood sample was divided into two parts. In one part, blood glucose concentration was immediately measured by a GEM Premier 3000 blood gas/glucose analyzer. During the study period, maintenance, calibration, and quality control of this blood gas/glucose analyzer was performed on a regular basis by the central hospital laboratory. The other part of blood sample was immediately sent to the central laboratory in a serum-separating tube (BD Vacutainer® SSTTM II Advance, LOT: 1266616, Becton Dickinson and Company, Plymouth, UK). After centrifugation, the serum glucose concentration was measured by a HITACHI 7600-020 biochemical analyzer (Hitachi High-Technologies, Tokyo, Japan) with an oxygen electrode oxidation method. At the same time of blood sampling, glucose value on CGMS monitor was also documented by one of the investigators. For glucose measurement, each data set at one time point contained three simultaneous glucose measurements: serum glucose concentration measured by CLD, whole venous blood glucose concentration measured by GEM Premier 3000 blood gas/glucose analyzer, and subcutaneous interstitial glucose concentration monitored by CGMS.

The alarm of CGMS was turned off to avoid interruption to bedside physicians and nurses' clinical decision. Although bedside nursing and physician teams were aware of patient's enrolment, they did not assess CGMS readings and change the patient management according to values from the CGMS.

### Statistical analysis

Statistical analyses were carried out by using MS Excel for MAC (Microsoft Corporation, Beijing, China) and SPSS statistical software (version 10.0, SPSS, Chicago, IL). Categorical variables were expressed as percentages. Continuous data were checked for normal distribution by Kolmogorov-Smirnov test, and were shown as mean and standard deviation (SD) or median with the 25th and 75th percentiles, when applicable.

By using the standard serum glucose concentration measured by CLD as reference, the accuracy of glucose measurement by CGMS or GEM Premier 3000 blood gas/glucose analyzer was analyzed. The numerical accuracy of measurements was evaluated by calculating relative absolute difference (RAD: absolute difference between time-matched measurement and reference divided by reference value, multiplied by 100), and by Bland and Altman's limits of agreement analysis [30]. Bias was defined as the mean of the difference between measurement and reference (measurement minus reference). Upper and lower limit of agreement were defined as bias±1.96 SD of the mean bias.

Clinical accuracy was evaluated by Clarke error grid analysis (Matlab R2011a, The Mathworks, Beijing, China) [31]. Results were divided into five zones: A, B, C, D, and E. Comparison points within zone A represent tested values that differ from the reference value by no more than 20%. Zone B includes comparison points that differ by more than 20%, but do not result in an alteration in treatment. Points in zone C would result in an overcorrection of acceptable glucose values. Points in zone D would result in failure to detect and treat errors. Comparisons in zone E would result in opposite treatment decisions. Values in zones A and B represent clinically accurate or acceptable results.

In order to clarify the influence of calibration time on the accuracy of CGMS, data sets were divided into those within 6 hours and those between 6 and 12 hours after CGMS sensor calibration. In order to determine the accuracy of CGMS at various glucose concentrations, data sets were evaluated over three different ranges for CLD serum glucose concentration: less than 3.9 mmol/L (hypoglycemia), 3.9 to 10.0 mmol/L (euglycemia), and greater than 10.0 mmol/L (hyperglycemia).

Categorical variables were analyzed by χ^2^ test. Comparisons of continuous data were performed by using unpaired t-test for normally distributed variables, and the Mann-Whitney U test for non-normally distributed variables. A p-value of less than 0.05 was considered statistically significant.

## Results

During study period, 684 patients were assessed for eligibility and 666 patients were excluded. The reasons for exclusion were admission only for overnight postoperative monitoring in 602 patients, blood glucose concentration less than 10.0 mmol/L at admission in 33 patients, younger than 18 years old in 19 patients, hemoglobin concentration less than 100 g/L in 9 patients, and in moribund and not likely to survive more than 24 hours in 3 patients. Finally, 18 patients were enrolled and 35 CGMS sensors were used during study period. Four sensors displayed malfunction and all occurred within the first 3 hours after sensor placement. Sensors were well tolerated in all patients. No serious adverse skin reactions, infections, or bleeding occurred during study period. All enrolled patients received insulin infusion during the study period, and no patient died. Demographic data of patients are shown in Table 1. In total, 314 glucose measurement data sets were obtained for analysis.

**Table 1:** Demographic data of enrolled patients.

Number of patients	18
Age (years)	58.4±13.1
Male	55.6%
History of diabetes mellitus	27.7%
Blood glucose concentration at admission (mmol/L)	14.24±2.36
APACHE II score	20.8±3.6
Time of CGMS monitoring per patient (hours)	83.3±36.1
Reason for ICU admission	
Severe traumatic brain injury	7 (38.9%)
Subarachnoid hemorrhage	4 (22.2%)
Acute lung injury after craniotomy	4 (22.2%)
Acute lung injury after chest trauma	3 (16.7%)

Data are mean ± SD, or n (%) unless otherwise stated.

### Accuracy of real-time CGMS

**Figure 1:**
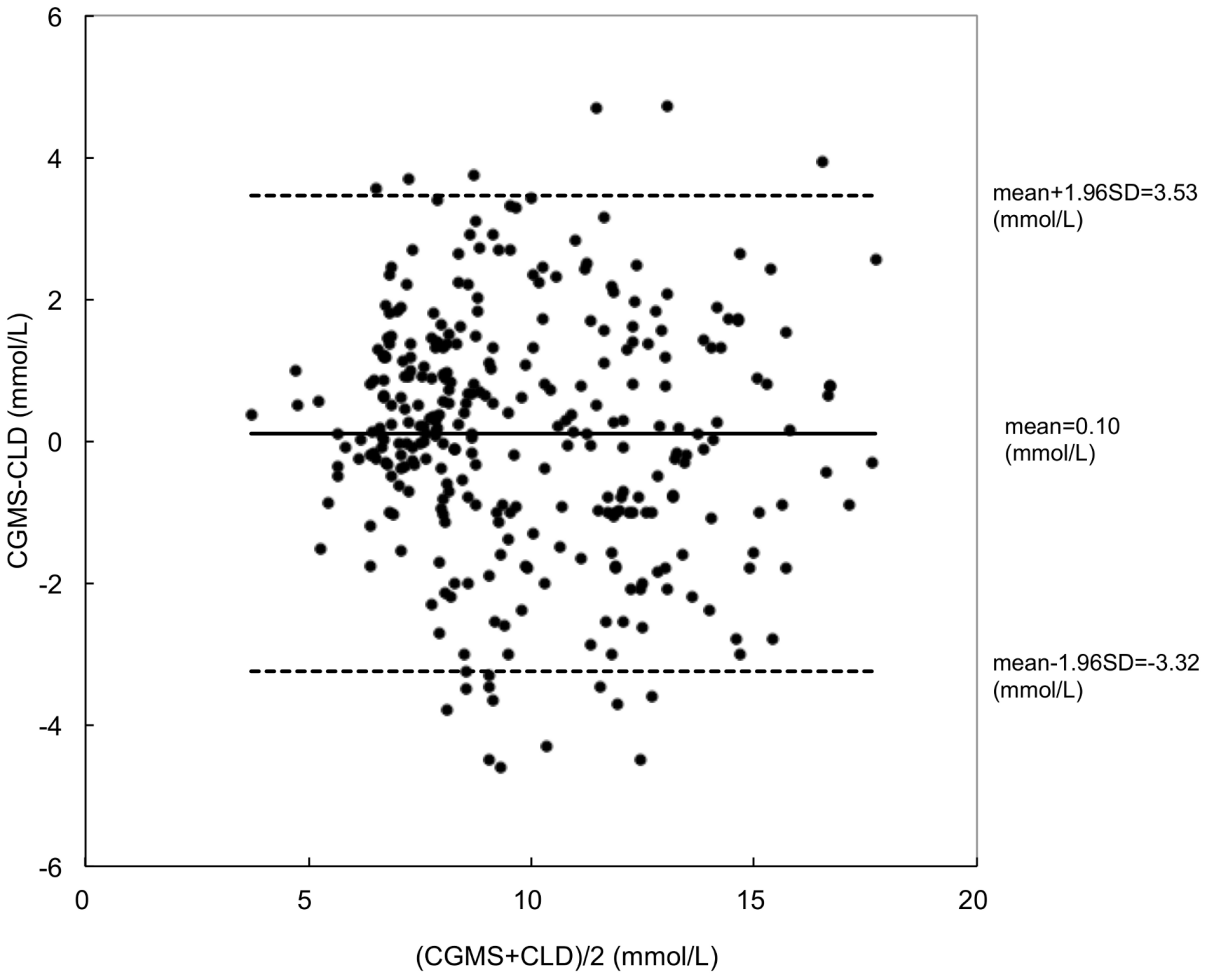
Bland and Altman plot between CGMS and CLD values. Differences between individual time matched CGMS and CLD values (y-axis) are plotted against means of time matched values (x-axis). Bias (solid line) and upper and lower limits of agreement (dashed line) are also displayed. CGMS = continuous glucose monitoring system. CLD = central laboratory device.

**Figure 2:**
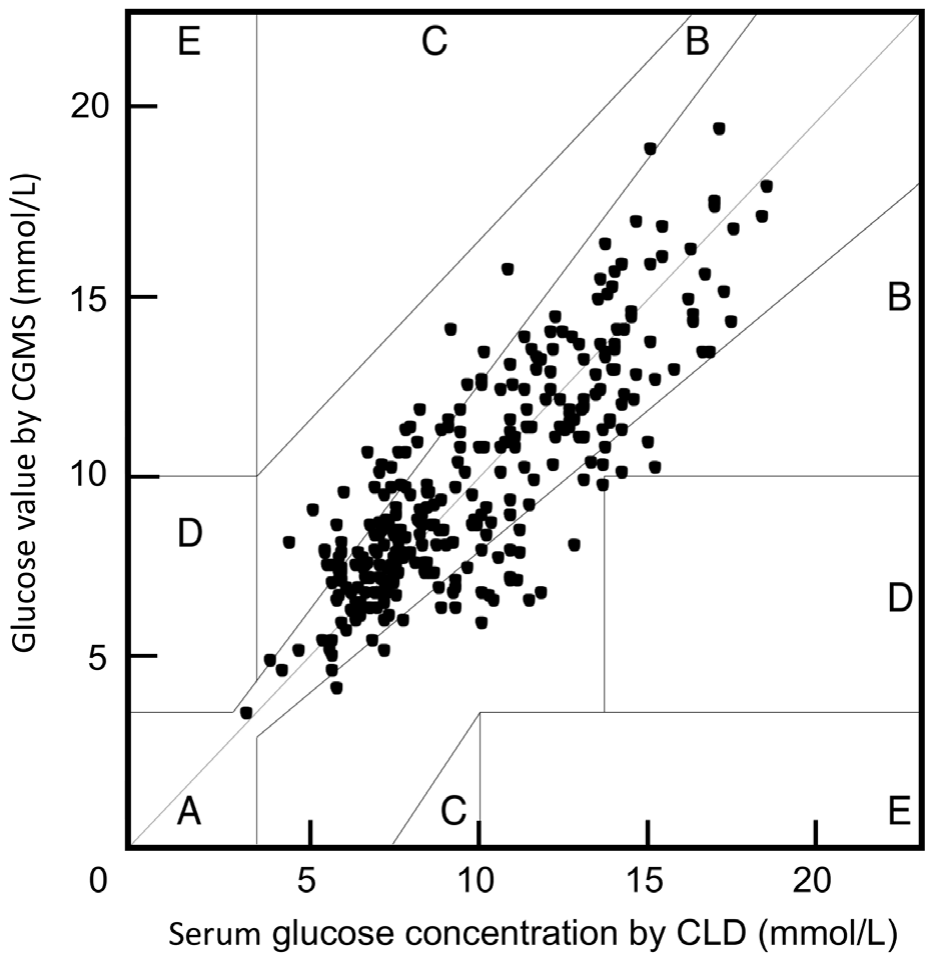
Clarke error grid analysis for comparison points between CGMS and CLD values. Comparison points were divided into five zones: A, B, C, D, and E. Comparison points within zone A represent tested values that differ from the reference value by no more than 20%. Zone B includes comparison points that differ by more than 20% but do not result in an alteration in treatment. Points in zone C would result in an overcorrection of acceptable glucose values. Points in zone D would result in failure to detect and treat errors. Comparisons in zone E would result in opposite treatment decisions. Values in zones A and B represent clinically accurate or acceptable results. There are 74.8% CGMS and CLD matched points in zone A and 25.2% in zone B, and no values in zone C, D or E. CGMS = continuous glucose monitoring system. CLD = central laboratory device.

With serum glucose concentration measured by CLD as reference, the RAD (mean±SD) between CGMS and CLD measurements was 14.4%±12.2%. Bland and Altman plot is shown in Figure 1. Bias and upper and lower limits of agreement between CGMS and CLD values were 0.10, 3.46, and −3.25 mmol/L, respectively (Figure 1). In Clarke error grid analysis, there were 74.8% matched points in zone A and 25.2% in zone B, and no values in zone C, D or E (Figure 2).

### Accuracy of real-time CGMS at different time after sensor calibration

The RAD between CGMS and CLD measurements in data sets obtained within 6 hours after sensor calibration (8.8%±7.2%) was significantly lower than those obtained between 6 to 12 hours after calibration (20.1%±13.5%, p<0.0001, Table 2). Bias and limits of agreement are also shown in Table 2. There was no significant difference in bias (p = 0.199). The percentage of matched points in zone A of error grid analysis was significantly higher in data sets within 6 hours after calibration than that between 6 to 12 hours after calibration (92.4% versus 57.1%, p<0.0001, Table 2).

**Table 2:** RAD, limits of agreement analysis, and Clarke error grid analysis between CGMS and CLD values in data sets at different time after CGMS sensor calibration and in different glycemic ranges.

	Data sets at different time after senor calibration			Data sets in different glycemic ranges		
	Within 6 hours	Between 6 to 12 hours	p	Euglycemic levels	Hyperglycemic levels	p
Number of data sets	158	156		176	137	
RAD (mean±SD)	8.8%±7.2%	20.1%±13.5%	<0.0001	15.4%±13.8%	13.2%±9.8%	0.228
Bias (mmol/L)	−0.02	0.23	0.199	0.65	−0.60	<0.0001
Upper and lower limits of agreement (mmol/L)	2.08 to −2.12	4.49 to −4.03		3.26 to −1.96	3.11 to −4.31	
Percentage of matched points in Clarke error grid						
Zone A	92.4%	57.1%	<0.0001	69.9%	81.0%	0.026
Zone B	7.6%	42.9%		30.1%	19.0%	
Zone C, D, and E	0	0		0	0	

Comparisons of RAD and bias were performed by unpaired t-test. Difference in percentage of matched points in Clarke error grid zone A was analyzed by χ^2^ test. RAD was calculated as absolute difference between time-matched CGMS and CLD value divided by CLD value, multiplied by 100. Bias was defined as the mean of the difference between time-matched CGMS and CLD values (CGMS minus CLD). Euglycemic level was defined as CLD serum glucose concentration of 3.9 to 10.0 mmol/L, and hyperglycemic level was defined as CLD serum glucose concentration greater than 10.0 mmol/L. Upper and lower limit of agreement were defined as bias±1.96 SD of the mean bias. CGMS = continuous glucose monitoring system. CLD = central laboratory device. RAD = relative absolute difference.

### Accuracy of real-time CGMS at different serum glucose concentrations

In total of 314 data sets, only one had CLD serum glucose value less than 3.9 mmol/L (3.5 mmol/L). In this data set, corresponding CGMS value was 3.9 mmol/L. Data of RAD and limits of agreement in euglycemic (3.9 to 10.0 mmol/L) and hyperglycemic levels (greater than 10.0 mmol/L) are shown in Table 2. There was no significant difference in RAD between the two different serum glucose levels (15.4%±13.8% in euglycemia and 13.2%±9.8% in hyperglycemia, p = 0.228). However, Bias in hyperglycemic levels was significantly more negative than that in euglycemic levels (p<0.0001). The percentage of matched points in zone A of Clarke error grid analysis was significantly higher in data sets in hyperglycemic levels than that in euglycemic levels (81.0% versus 69.9%, p = 0.026, Table 2). When analysis was performed only in data sets obtained within 6 hours after sensor calibration, no significant differences were found either in RAD (8.8%±7.2% versus 8.7%±7.3%, p = 0.950) or percentage of matched points in Clarke error grid zone A (90.6% versus 95.1%, p = 0.370) between different glucose levels. Bias in hyperglycemic levels was also significantly more negative than that in euglycemic levels (0.16 versus −0.32 mmol/L, p = 0.006).

### Accuracy of ICU-based GEM Premier 3000 blood gas/glucose analyzer (GEM)

With serum glucose concentration measured by CLD as reference, the RAD between GEM and CLD measurements was 6.5%±6.2%. Bias and upper and lower limits of agreement between GEM and CLD values were −0.26, 1.35, and −1.87 mmol/L, respectively. In Clarke error grid analysis, there were 98.4% matched points in zone A and 1.6% in zone B, and no values in zone C, D or E.

## Discussion

Real-time glucose monitoring has been advocated for many years to improve glycemic management in critical care settings [6], [27]. In present study, the accuracy of a real-time subcutaneous CGMS was assessed in glucose monitoring in critically ill patients. An acceptable accuracy was found, either numerically or clinically, for subcutaneous CGMS in real-time glucose monitoring. Most importantly, CGMS showed highly accurate within 6 hours after sensor calibration. Different methods and time intervals for CGMS sensor calibration have been employed by studies in ICU settings, and this may contribute to the disparity in results in accuracy evaluation. Sensors were calibrated against capillary, arterial, and venous blood glucose measurement by POC handheld glucose analyzers [14], [17], [18], [20]–[23], [25], [26], or against arterial blood glucose values by ICU based blood gas/glucose analyzers [11], [15], [16], [19], [24]. Sensor calibration was performed every 6 hours in majority of studies [11], [14]–[16], [19], [23], [24], whereas 12-hour time interval [18], [20], [21] and before each meal [22] were chosen in other studies. Up to now, no study has been carried out to investigate the influence of calibration time on accuracy of CGMS. According to recommendation by the manufacturer, we calibrated sensors in 12-hour interval. Although CLD value is considered the “gold standard” for blood glucose measurement, it is not suitable for simultaneous CGMS sensor calibration because of its slow turn-around time. Because glucose measurement by ICU-based blood gas/glucose analyzer has been proven to be more accurate than that by POC handheld glucose analyzers [11], [12], we finally chose venous glucose concentration measured blood gas/glucose analyzer as GCMS calibration method. With CLD measurement as reference, our results for numerical agreement and Clarke error grid analysis are comparable to those studies with 12-hour calibration interval [18], [20], [21]. Furthermore, the results from data sets within 6 hours after calibration showed a higher accuracy, with 8.8%±7.2% of RAD and 92.4% of matched points in Clarke error grid zone A (Table 2). These results are similar to those studies in critically ill patients by Corstjens et al [11] and Brunner et al [24]. Both of the studies employed blood gas/glucose analyzer and 6-hour interval for CGMS sensor calibration. Our study indicates that, no matter what time interval for sensor calibration recommended by manufacturer, CGMS should be calibrated shorter than 6 hours. However, although over 300 paired samples were analyzed in present study, we only enrolled 18 patients in a single ICU. So the results of our study may not be generalizable to other critically ill populations. Further studies are needed in the field of real-time glucose monitoring.

There was only one CLD glucose measurement below 3.9 mmol/L during the study period. This may be contributed to the fact that we only enrolled patients with hyperglycemia at admission and we employ a conventional glucose control protocol in our routine clinical practice. For comparison between euglycemia and hyperglycemia, although RAD was not significantly different in this two glucose levels, limits of agreement between CGMS to CLD measurements was significantly more negative in hyperglycemia (bias = −0.60 mmol/L, upper and lower limits of agreement = 3.11 to −4.31 mmol/L) than that in euglycemia (bias = 0.65 mmol/L, upper and lower limits of agreement = 3.26 to −4.31 mmol/L), even in data sets within 6 hours after sensor calibration. This might be explained by the lag of change for interstitial glucose concentration to serum glucose concentration [32]. Although this bias was clinical acceptable, danger of underestimation of hyperglycemia by CGMS also existed. To avoid this bias, reference measurement, such as ICU-based blood gas/glucose analysis, should be performed when CGMS monitoring exhibits abrupt change.

Blood gas analyzer with function of glucose measurement is one of the most frequently used glycemic monitoring methods in ICU settings [3], [28], [33]. Most of ICUs in China have been equipped with this kind of instrument, and measurement of blood glucose has become routine care in these ICUs [34]. Several studies have been carried out to determine the accuracy of blood gas/glucose analyzers in blood glucose measurement [11], [12]. In present study, we used deep venous blood in glucose measuring by an ICU-based gas/glucose analyzer. With serum glucose concentration measured by CLD as reference, blood gas/glucose analyzer measurements show a pretty good numerical and clinical accuracy, with 6.5%±6.2% of RAD and 98.4% of points in Clarke error grid zone A. This result indicates that the ICU-based blood gas/glucose analyzer is an accurate alternative for CLD glucose measurement, which can serve as a standard method for glucose monitoring and CGMS sensor calibrating.

The major limitation in our study is that there were too few glucose data below 3.9 mmol/L to investigate the accuracy of CGMS monitoring in hypoglycemic state. After publication of NICE-SUGAR study [28], many physicians in our ICU are concerned about hypoglycemia during tight glucose control, and therefore to employ a conventional glucose control protocol in clinical practice. For further study, more patients are needed to increase the chance of hypoglycemia for CGMS accuracy investigation. In order to prevent the interruption of CGMS reading on routine clinical care, we turned off alarm of CGMS during the study. The main theoretical usefulness of CGMS is its ability to detect glucose concentration variation and give alert. Further study is needed to investigate the accuracy of CGMS during change of glucose indicated by preset alarms.

## Conclusions

Real-time subcutaneous CGMS is an accurate glucose monitoring method in critically ill patients. CGMS sensor should be calibrated less than 6 hours, no matter what time interval recommended by manufacturer.
